# Adrenal hemorrhage following direct oral anticoagulant (DOAC) therapy: two case reports and literature review

**DOI:** 10.1186/s12959-022-00397-9

**Published:** 2022-07-05

**Authors:** Elahe Sheklabadi, Yasaman Sharifi, Mahdi Tabarraee, Seyed Saeed Tamehrizadeh, Parham Rabiee, Farzad Hadaegh

**Affiliations:** 1 Prevention of Metabolic Disorders Research Center, Research Institute for Endocrine Sciences, Shahid Beheshti University of Medical Sciences, No. 24, Parvaneh Street, Velenjak, P.O. Box: 19395 − 4763, Tehran, Iran; 2 Department of Radiology, School of Medicine, Iran University of Medical Sciences, Tehran, Iran; 3 Department of Hemato-Oncology, Taleghani Hospital, Shahid Beheshti University of Medical Sciences, Tehran, Iran; 4 Rajaie Cardiovascular, Medical and Research Center, School of Medicine, Iran University of Medical Sciences, Tehran, Iran

**Keywords:** Adrenal hemorrhage, Direct oral anticoagulants, Autoimmune Addison disease, Adrenal insufficiency, APS-2

## Abstract

**Background:**

Adrenal hemorrhage (AH) is a rare condition that can result in a life-threatening medical emergency. This medical condition could be caused by several underlying factors, one of which is the use of anticoagulants. As far as we are aware, direct oral anticoagulant (DOAC) agents are a rare but possible cause of AH.

**Case presentation:**

Herein, we described two cases of AH due to DOACs. The first case was a 35-year-old Iranian woman with a past medical history of Hashimoto thyroiditis who was being treated with apixaban due to the previous thrombosis. Her first symptoms of AH (November 2021) were strangely similar to symptoms of autoimmune Addison disease (AAD) which led to a confirmed diagnosis of autoimmune polyendocrine syndrome type 2 (APS-2). An abdominal MRI revealed an oval shape well-encapsulated cystic mass with a diameter of 20 × 14 mm with a thick and low signal intensity rim in the left adrenal gland, highly suggestive of sub-acute left-sided AH. Our second case was an 89-year-old Iranian woman who had been admitted to the hospital (August 2021) with low blood pressure and disorientation. At the beginning of her admission, the evaluation showed hyponatremia, and further evaluations confirmed adrenal insufficiency (AI). The patient reported rivaroxaban usage for deep vein thrombosis prophylaxis after femur fixation surgery. Her abdominal CT scans showed bilateral adrenal masses highly suggestive of AH. Her follow-up examination showed persistent AI after three months.

**Conclusion:**

Given the history of our cases, physicians should be aware of AH in patients receiving DOACs, particularly in elderly patients who are at high risk of bleeding. It is also worth noting that AH can occur in any patient with any medical history and history of DOAC use, which is why patients must be closely monitored.

## Background

Adrenal hemorrhage(AH) is considered a rare but potentially lethal condition that can occur in both traumatic and non-traumatic conditions [1]. Formerly, AH was mostly diagnosed during post-mortem examinations, with incidence rates ranging from 0.14% to 1.1% [1–4]. Due to tremendous advancements in imaging technology, AH is now diagnosed with a higher frequency in hospitalized patients, ranging from 1.5% to 5% [1, 5, 6].

Factors that may lead to AH include focal adrenal lesions, abdominal trauma, anticoagulation therapy, congenital or acquired bleeding disorders, sepsis, and pregnancy [1, 7, 8]. AH can be presented in asymptomatic settings to fatal conditions, mostly resulting from bilateral AH [8, 9]. It has been revealed that bilateral AH through adrenal insufficiency (AI) induction can be detrimental. A key difference between unilateral and bilateral AH is that the former is mostly biochemically silent, while the latter is potentially fatal [10, 11].

Anticoagulation options have rapidly expanded over the last decade, with the United States Food and Drug Administration (FDA) approval of the first direct oral anticoagulant (DOAC) in 2001, rivaroxaban in 2011, apixaban in 2012, and edoxaban in 2015 [12, 13]. Compared to vitamin K anticoagulants (VKA), DOACs have fewer interactions with medications and diet, have a more stable pharmacokinetics profile, and do not require routine laboratory monitoring. These findings led to the replacement of DOACs with VKA [14].

Herein, we reported for the first time a case of unilateral AH attributed to apixaban treatment in a newly diagnosed patient with previously confirmed autoimmune Addison disease (AAD) and the second case of bilateral AH following administration of rivaroxaban in an elderly woman.

## Case presentation

### Case 1

A 35-year-old Iranian woman presented with anorexia, nausea, and non-bloody frequent vomiting 2 months ago referred to a gastroenterologist in November 2021. She was complaining of fatigue, tiredness, postural dizziness, salt craving, dry skin, progressive buccal pigmentation, memory impairment, and about 5 kg of weight loss in 2 months. The patient denied pain in her abdomen, flank, or chest. No history was reported for abdominal blunt trauma. Medical history revealed hypothyroidism in the background of Hashimoto’s thyroiditis from four years ago. Additionally, the patient had a history of right internal jugular vein thrombosis (IJVT) eight years ago that was attributed to oral contraception pill consumption and was treated with warfarin for about one year. Moreover, the patient reported left IJVT last year which occurred two weeks after the coronavirus disease of 2019 (COVID-19) infection.

She has been under treatment with levothyroxine 350 μg per week and apixaban 2.5 mg twice a day since one year ago. She was married with no children. Her menstruation was regular. Her family history was unremarkable, excluding her sister’s hypothyroidism.

**Fig. 1:**
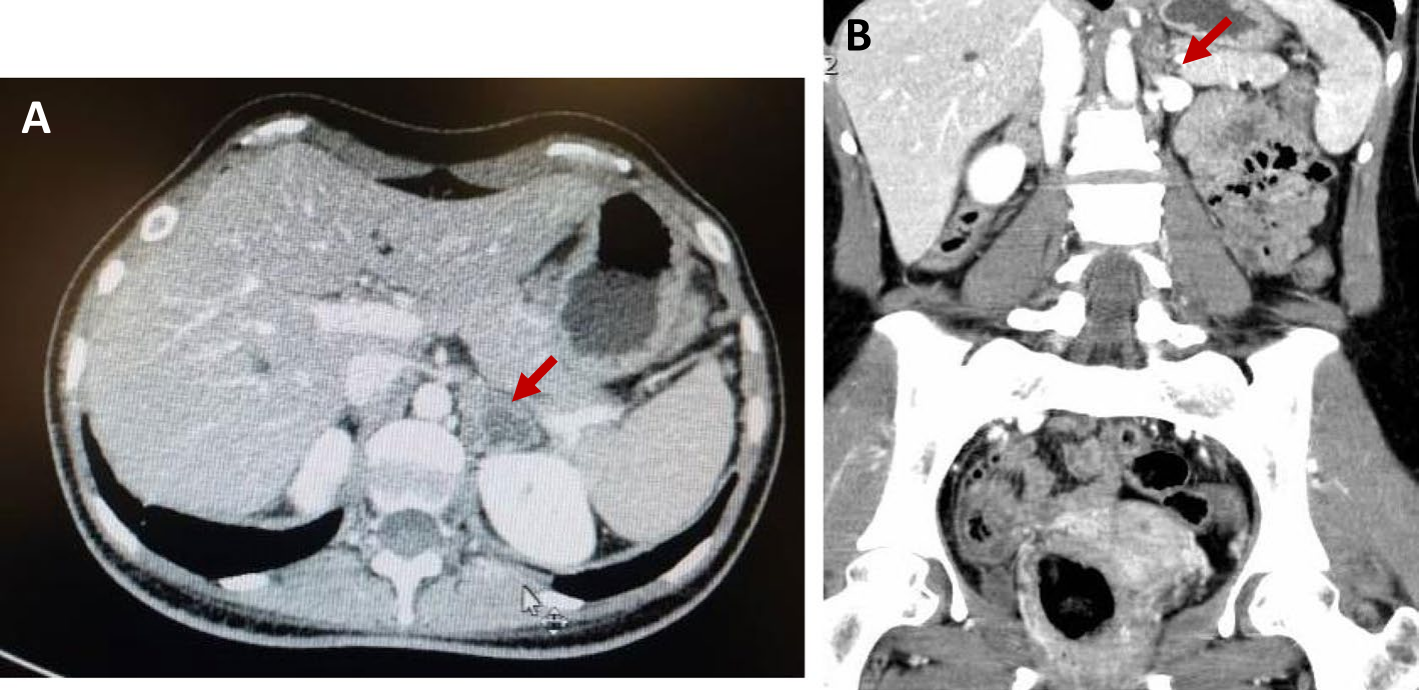
A computed tomography scan of the abdomen and pelvis of the first case with contrast shows left adrenal thick wall cystic mass with an enhanced peripheral rim. (**A**) The transverse view and (**B**) the coronal view. The red arrows indicate the areas of fat stranding

Due to her gastrointestinal symptoms and weight loss, an abdominopelvic computed tomography (CT) scan with contrast has been requested. CT showed a 32 × 22 mm left adrenal thick wall cystic mass with an enhanced peripheral rim. The right adrenal gland was normal (Fig. 1). Hence the patient was admitted to the hospital.

On physical examination, systolic blood pressure (SBP):85 mmHg, temperature(T): 37 °C, pulse rate (PR): 80 beats/min. body mass index (BMI): 16.5 kg/m2. Skin: dry, pale, no vitiligo, no ecchymosis. Pigmentation in the buccal, gums, and tongue was found. Otherwise, the physical examination was unremarkable. Table 1 shows the results of lab data at the time of admission as well as the endocrinologist’s further workup.

**Table 1:** Laboratory parameters on the admission (November 2021) of our first case with unilateral adrenal hemorrhage in the background of autoimmune adrenal insufficiency in a 35 years old woman under treatment with apixaban

Parameters	Result	Reference range
Leukocytes(cells/cumm)	9300	3500–10,000
Hemoglobin (gr/dl)	10.7	12–16
Hematocrit (%)	32	34.7–46.7
MCV (fl)	87.5	81–100
Platelets(cells/cumm)	225,000	150,000–450,000
FBS (mg/dl)	77	65–100
BUN (mg/dl)	28	6–21
Cr (mg/dl)	1.2	0.5–1
Ca (mg/dl)	10	8.5–10.5
P (mg/dl)	5	2.5–4.5
Sodium(mEq/L)	134	132–145
Potassium (mEq/L)	4.4	3.6–5.2
ALT(IU/L)	24	5–32
AST (IU/L)	25	5–33
TSH (µIU/mL)	13.14	0.3–4.2
T3 (ng/mL)	1.08	0.5–1.5
T4(µg/dL)	5.9	5.1–14.1
Cortisol 8am (μg/dl)	1.1	6.2–20
ACTH (pg/ml)	> 1000	7.2–64
B12(pg/ml)	267.2	187–883
Folic Acid(ng/ml)	15.38	3.1–18.8
Anti TPO (IU/ml)	215.9	5–34
Ferritin (ng/ml)	217	13–150
Anti Tg (IU/ml)	347.7	Up to 50
TTG Ab (IgA) (RU/ml)	1.4	Neg < 10
VMA (mg/24 h urine)	8.4	2–21
Metanephrine (μg/24 h urine)	40.1	0–350
Normetanephrine (μg/24 h urine)	159.7	0–600
COVID-19 PCR	Negative	
PPD Test	Negative	

*MCV* mean corpuscular volume, *FBS* Fasting Blood Sugar, *BUN* blood urea nitrogen, *ALT* alanine transaminases = thyroid stimulating hormone, *ACTH* Adrenocorticotropic Hormone, *Anti TPO* Anti-thyroid peroxidase, *Anti Tg* Antithyroglobulin, *TTG Ab(IgA)* tissue transglutaminase antibody ( IgA), *VMA* Vanillylmandelic acid, *PPD* purified protein derivative

According to the history, physical examination, and presence of low cortisol levels (1.1 μg/dl) and high adrenocorticotropic hormone (ACTH) level, autoimmune Addison’s disease (AAD) was diagnosed. Hydrocortisone 150 mg per day was prescribed and clinical improvement occurred and BP level increased to 110/70 mmHg.

**Fig. 2:**
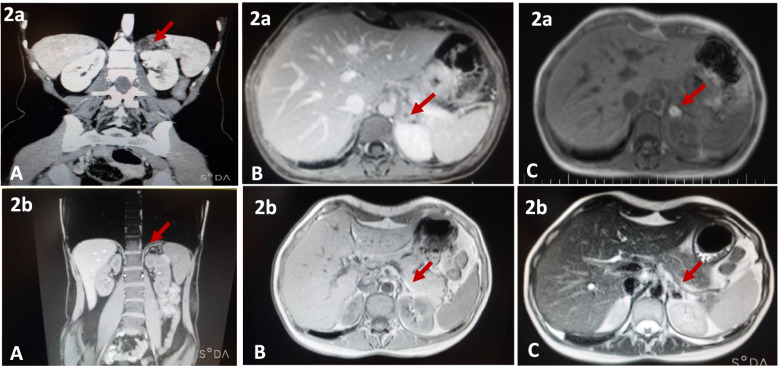
**a.** Abdominal magnetic resonance imaging (MRI) with gadolinium showed an oval shape well-encapsulated cystic mass with a thick and low signal intensity rim in the left adrenal gland(A: Coronal &B: axial view of hepatobiliary phase, C: hyperintense on T1 fat-saturated). The red arrows indicate adrenal hemorrhage. **b.** MRI with gadolinium showed near-complete resolution of left adrenal hematoma and residual hemosiderin is noted (A: Coronal & B: axial view of hepatobiliary phase, C: hyperintense on T1 fat-saturated)

An abdominal magnetic resonance imaging (MRI) with gadolinium was ordered to determine the exact diagnosis of the adrenal cystic lesion. The MRI showed an oval shape well-encapsulated cystic mass of 20 × 14 mm with a thick and low signal intensity rim in the left adrenal gland. This mass was high signal intensity on T1 and T2 weighted images, consistent with sub-acute hemorrhage. There was an enhancement of the peripheral thick wall rim after injection of contrast. The right adrenal gland was normal (Fig. 2a).

During the admission, the hemodynamics were stable. She was given prednisolone 7.5 mg daily and Fludrocortisone 0.05 mg daily. ACTH level decreased to 7.03 pg/ml after treatment. Levothyroxine was increased to 500 μg per week after AI management. Apixaban was discontinued, and she was referred to a hematologist for evaluation of thrombophilia and antiphospholipid syndrome (APLS). The hematological tests for APLS were requested but the patient refrained from performing the tests. Her follow-up laboratory tests after 3 months revealed an ACTH level of 86 pg/ml. As a follow-up, an adrenal MRI that was performed three months later revealed the disappearance of left adrenal hemorrhage and near-complete resolution of left adrenal hematoma (Fig. 2b).

### Case 2

An 89-year-old Iranian woman with a history of hypertension presented with fatigue, loss of appetite, and delirium, ten days after surgery to fix her neck femur in November 2020. She was using rivaroxaban 10 mg per day for deep vein thromboembolism prophylaxis. Her SBP dropped to 90 mmHg and the oral hypotensive agent (losartan 25 mg BID) was discontinued. No history of fever, head trauma, infection symptoms, or bleeding was reported. At the time of admission, the physical examination was SBP:90 mmHg, T:37 °C, PR:96 beats/min. BMI: 26.44 kg/m2. Her Mental status was disoriented. The rest of her physical examination was otherwise unremarkable.

Due to physical examinations and her disorientation, a brain MRI was requested that revealed evidence of subdural hematoma that was not progressive in the control MRI after 3 days. Initial laboratory tests are shown in Table 2. Considering the hypotension and hyponatremia the presence of Addison’s disease (AD) was suspected so cortisol at 8 am and ACTH level was requested that confirmed the diagnosis of AD, according to the cortisol level of 0.05 μg/dl and ACTH 212 pg/ml (Table 2). Hence, hydrocortisone was initiated for the patient and her clinical symptoms improved significantly. Her sodium level increased to 137 mEq/L. During the admission hemodynamics were stable.

**Table 2:** Laboratory parameters on the admission (August 2021) of our second case with bilateral adrenal hemorrhage in an 89 years old woman under treatment with rivaroxaban

Parameters	Result	Reference range
Leukocytes(cells/cumm)	15,100 (Neutrophil: 56%,Lymphocyte: 26%)	3500–10,000
Hemoglobin (gr/dl)	11.5	12–16
Hematocrit (%)	34.6	34.7–46.7
MCV (fl)	92.7	81–100
Platelets (cells/cumm)	259,000	150,000–450,000
BS (mg/dl)	90	65–100
BUN (mg/dl)	18	6–21
Cr (mg/dl)	0.7	0.5–1
Ca (mg/dl)	9.9	8.5–10.5
P (mg/dl)	3.7	2.5–4.5
Sodium (mEq/L)	126	132–145
Potassium (mEq/L)	5	3.6–5.2
ALT (IU/L)	49	5–32
AST (IU/L)	45	5–33
INR	2.2	1–1.3
PTT (sec)	36.4	28–45
TSH (μIU /ml)	1.7	0.3–4.2
T3(ng/ml)	1.1	0.5–1.5
T4(μg/dl)	9.02	5.1–14.1
Cortisol 8am (μg/dl)	0.05	6.2–20
ACTH (pg/ml)	212	7.2–64
B12(pg/ml)	874	187–883
Folic Acid(ng/ml)	15.1	3.1–18.8
Ferritin (ng/ml)	190.5	13–150
VMA (mg/24 h urine)	12	2–21
Metanephrine(μg/24 h urine)	37.9	0–350

*MCV* mean corpuscular volume, *BS* Blood Sugar, *BUN* blood urea nitrogen, *ALT* alanine transaminases, *AST* Aspartate aminotransferase *INR* international normalized ratio, *PTT* Partial thromboplastin time, *TSH* thyroid stimulating hormone, *ACTH* Adrenocorticotropic Hormone, *VMA* Vanillylmandelic acid

**Fig. 3:**
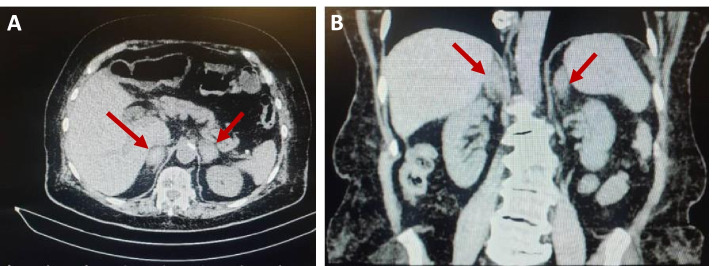
A computed tomography scan of the abdomen and pelvis of the second case shows bilateral adrenal masses with the same features on right and left sides with high density in non-contrast images. (**A**: axial view and **B**: Coronal view). The red arrows indicate adrenal hemorrhage

Adrenal CT scans with and without contrast were performed to determine the cause of AI. As shown in Fig. 3 bilateral adrenal masses were found as 38 × 25 mm on the right side and 35 × 26 mm on the left side with high density in non-contrast images (Hounsfield unit (HU):40–50), without enhancement in the portal and delayed phases, more likely to be intra-adrenal hemorrhage. Due to her AH and subdural hematoma on her MRI, the rivaroxaban has been discontinued. She has been discharged with prednisolone 7.5 mg per day and fludrocortisone 0.05 mg per day.

**Fig. 4:**
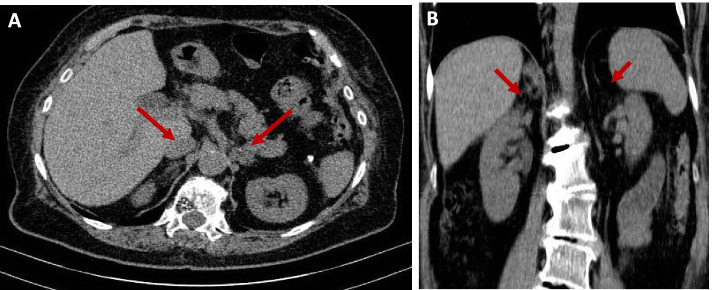
Follow-up Computed tomography scan of the abdomen and pelvis of the second case after three months showed a stable size and decreasing density on both sides with a mean HU < 10 in non-contrast images. (**A**: axial view and **B**: Coronal view). The red arrows indicate adrenal hemorrhage

Follow-up adrenal CT, three months later, showed decreased size in both adrenal hemorrhages with a mean HU < 10 (Fig. 4). Her follow-up laboratory tests after 3 months revealed that her AI had persisted, with a Cortisol 8 am level of 0.4 μg/dl and an ACTH level of 204 pg/ml.

## Search strategy for literature review

In a PubMed search, we searched for articles published between January 2000 and February 2021 containing the keywords "autoimmune Addison disease and adrenal hemorrhage", "DOAC".

treatment and adrenal hemorrhage", "adrenal insufficiency and adrenal hemorrhage", and "AH and long-term AI as well as "AH and autoimmune polyendocrine syndrome type 2". A comprehensive review of all relevant studies was conducted, and all references were manually checked for additional studies that may have been relevant.

## Discussion

Here, we report two cases of AH post-DOAC prophylaxis. It is a novel case of unilateral AH caused by apixaban usage in a patient with newly diagnosed AAD. An APS-2 diagnosis was made that included Hashimoto hypothyroidism as well. In another case of bilateral AH after prophylactic rivaroxaban usage, the patient developed AI, which persisted during 3 months follow-up.

AH is a rare medical emergency, with an incidence rate of 0.14%-1.1% reported in some of the largest (> 25,000 cases) autopsy series [1–4, 15]. Several underlying stress conditions can cause non-traumatic AH, such as sepsis, pregnancy, post-abdominal surgery, anti-phospholipid syndrome, and, in rare cases, anticoagulation therapy [2, 16–20]. Bleeding is a potential risk associated with every anticoagulant medication and is the most common side effect of these medications, leading to hospitalizations and deaths [12, 21].

The most similar case to ours in the search for the etiology of AH is an article by Zachary Sanford et al. [22]. It described a case of AH in a patient with APLS who had previously been on warfarin prophylaxis, with reoccurring AH presented with right flank pain followed by retinal hemorrhage precipitated during a transition from warfarin to apixaban. It is noteworthy that this is the only case reported in which a unilateral AH occurred in a patient receiving apixaban anticoagulation [22]. We reported here the second unilateral AH due to apixaban use that was accidentally detected in CT of adrenal glands in a newly diagnosed patient with AAD and probable adrenocortical atrophy.

Clinical suspicion and prompt diagnosis of bilateral AH are clinically vital because 16–50% of patients with bilateral AH eventually develop life-threatening AI [1, 23]. CT scans with and without IV contrast or MRI with Gadolinium are commonly used to diagnose AH [24, 25].MRI is more accurate than other imaging modalities in diagnosing adrenal hematoma, and it may also differentiate between subacute and chronic hemorrhage [25, 26]. In our case with AAD, Hashimoto thyroiditis, and APS-2, the patient had left adrenal thick wall cystic mass with the enhanced peripheral rim on CT scan imaging, which suggests adrenal masses or AH. MRI findings in our case include an oval shape well-encapsulated cystic mass 20 × 14 mm with a thick and low signal intensity rim in the left adrenal gland. This mass was high signal intensity on both T1 and T2 weighted images, the findings suggestive of sub-acute AH [27].

Primary AI is the result of partial or complete bilateral adrenal cortex destruction leading to deficiency of all adrenocortical hormones (also known as AD). Tuberculosis was the most common cause of AD in the first half of the twentieth century, but AAD has recently overtaken it[23]. In patients with APLS, AH is a relatively uncommon cause of AD as a result of bilateral AH [7, 23, 28].

Diagnosis of AH is difficult due to the nonspecific clinical presentation. Therefore, having a high level of clinical suspicion plays a crucial role in facilitating an earlier diagnosis and avoiding undesirable outcomes. These signs and symptoms include pain in the back or flanks, nausea, vomiting, hypotension, and fever [29–31]. Our patient with AAD and unilateral AH had no symptoms related to unilateral AH excluding loss of appetite, nausea, vomiting, and buccal hyperpigmentation, which were more likely to be caused by deficiency of adrenocortical hormones. To the best of our knowledge, no other case of AAD associated with autoimmune polyendocrine syndrome type 2 (APS-2) before AH diagnosis has been reported. APS-2 is the most common autoimmune polyendocrine syndrome [32]. A hallmark of this condition is its combination of autoimmune AD with thyroid autoimmune disease and/or type 1 diabetes mellitus [32]. Similar to our first case, AD and Hashimoto thyroiditis (Schmidt syndrome) are the most common clinical combinations [32]. The presence of 21-hydroxylase autoantibodies (21OHAb), is used as a confirmation test for the diagnosis of AAD [33]. One of the limitations of our study is the country's limited access to antibody tests, which made it difficult to confirm the diagnosis based on laboratory autoimmune tests.

Due to their stable pharmacokinetic profile and fewer interactions with other medications and diets, DOACs currently do not require routine laboratory monitoring. As a result, DOACs have supplanted VKA as the first-line anticoagulant in international guidelines for managing and preventing thrombotic diseases [12–14, 34]. Because routine coagulation tests cannot be used to determine the degree of anticoagulation in individuals receiving a DOAC, managing bleeding can be challenging [12].To the best of our knowledge, only five cases of AH have been reported as a result of the use of the latest anticoagulant drugs (Table 3). Four with the use of rivaroxaban [10, 35–37], and only one through the use of apixaban [22] prophylactic treatment. However, all of the evaluated cases demonstrated AI as a result of bilateral AH. Although bilateral AH is more commonly associated with AI, the contralateral adrenal gland may become "exhausted" in unilateral AH [15]. This may result in hypocortisolemia due to decreased cortical lipids, as demonstrated in a case presented by B.Ly [37], a case of unilateral AH with confirmed AI in the laboratory test. Two of these four were under prophylactic anticoagulation following knee surgery in the patients [10, 37], and the rest were due to APLS [35, 36]. Our second case was similar to the mentioned cases because AH also occurred following rivaroxaban treatment after femur fixation surgery. In addition, she had bilateral AH, which could lead to AI.

**Table 3:** Demographics, presentation, the type of anticoagulant drugs and the presence of Addison disease in current cases and literature reviews of AH due to DOACs usage. NM = not mentioned, APLS = antiphospholipid syndrome, IJVT=internal jugular vein thrombosis, AH = adrenal hemorrhage, AI = adrenal insufficiency

No	Author, [reference]	Age/Gender	Presentation	Anticoagulant drugs	Anticoagulant drugs dosage	Reason to get Anticoagulant prophylaxis	Laterality of AH	Presence of Addison Disease as a medical background	AI secondary to the AH
1	W. Comuth(2017) [36]	63/Female	Abdominal pain	Rivaroxaban (Xarelto)	20 mg daily	APLS	Bilateral	-	+
2	Z. Sanford(2019) [22]	42/Male	Acute onset severe right flank pain	Apixaban (Eliquis)	NM	APLS	Unilateral	-	-
3	B. A. Ly(2019) [37]	61/Male	Nausea and vomiting	Rivaroxaban	10 mg daily	Knee Surgery	Unilateral	-	-
4	M. Alidoost(2019) [10]	68/Female	Severe acute onset abdominal pain	Rivaroxaban	10 mg daily	Knee Surgery	Bilateral	-	+
5	M. A. Arosemena(2020) [35]	46 / Male	Syncopal episode	Rivaroxaban	NM	APLS	Bilateral	-	+
6	Current Case(2022)	35/Female	Nausea and vomiting and buccal hyperpigmentation	Apixaban	2.5 mg twice a day	IJVT	Unilateral	+	+
7	Current Case(2022)	89/Female	Fatigue and loss of appetite	Rivaroxaban	10 mg daily	Femur Surgery	Bilateral	-	+

DOACs have shown equivalent or greater efficacy and safety compared to vitamin K agonists or enoxaparin in frail or elderly subjects and after orthopedic surgeries [12]. However, because DOAC antidotes (idarucizumab for reversal of dabigatran, and andexanet alfa for reversal of the direct FXa inhibitors apixaban and rivaroxaban), are not available in our country, the elderly woman with a femoral neck fracture, was not a good candidate for DOAC agents; instead, warfarin or low molecular weight heparin (LMWH) agents with available antidotes might be a better prophylactic treatment [13, 21].

In patients with bilateral AH, the long-term follow-up of glucocorticoid and mineralocorticoid function has been assessed in only a few articles [38–43]. After a follow-up of four patients up to 19 years with acute bilateral AH and glucocorticoid insufficiency, the researchers showed the absence of need for long-term mineralocorticoid replacement. They also demonstrated the improvement in serum cortisol levels in three of the four patients and the ability of one case to function normally without cortisol replacement for 4 years. In our second case with bilateral AH, we found the need for continued adrenocortical replacement therapy after three months of follow-up.

As a limitation, we did not perform an adrenocorticotropic hormone stimulation test in our cases for confirmation of AI, however, according to the guideline released by “Endocrine Society Clinical Practice Guideline “If a corticotropin stimulation test is not feasible, using morning cortisol < 5 μg/dL in combination with high ACTH ( i.e. > twofold upper limit of the reference range) is suggestive of adrenal insufficiency [44]. Moreover, as suggested by many authors [23, 45] morning cortisol concentrations lower than 3 μg/dL are strongly predictive of adrenal insufficiency. In both of our cases with AI, the levels of cortisol were less than 1.5 μg/dL and the value of ACTH levels were more than 3 folds of the upper limits of the normal range.

In the current study, we presented two cases of AH caused by DOAC use. The first unilateral AH occurred after the therapy with apixaban in a newly diagnosed patient with AAD and the second one was a bilateral AH that occurred after the prophylactic therapy with rivaroxaban following femur fixation surgery. Any patient with any medical background might adversely develop side effects like AH when treated with DOACs, even patients that are low risk such as the young 35-year-old patient that was presented in case 1. probable adrenocortical atrophy.

## Data Availability

Data sharing does not apply to this article as no datasets were generated or analyzed during the current study.

## Abbreviations

AH: Adrenal hemorrhage
DOAC: Direct oral anticoagulant
AAD: Autoimmune Addison disease
APS-2: Autoimmune Polyendocrine Syndrome type 2
AI: Adrenal Insufficiency
VKA: Vitamin K Anticoagulants
IJVT: Internal jugular vein thrombosis
COVID-19: Coronavirus Disease of 2019
CT: Computed Tomography
SBP: Systolic Blood Pressure
T: Temperature
PR: Pulse Rate
BMI: Body Mass Index
ACTH: Adrenocorticotropic Hormone
MRI: Magnetic resonance imaging
HU: Hounsfield unit
APLS: Antiphospholipid syndrome
AD: Addison disease
LMWH: Low molecular weight heparin
